# Valorization of Volcanic Ash and Stainless Steel Slag as Partial Replacements of Metakaolin in Geopolymer Binders

**DOI:** 10.3390/ma19040719

**Published:** 2026-02-13

**Authors:** Youssef Ettahiri, Raúl Vico Lujano, Lahcen Bouna, Abdeljalil Benlhachemi, José Miguel Cáceres-Alvarado, Dolores Eliche-Quesada, Luis Pérez-Villarejo

**Affiliations:** 1 Materials and Environment Laboratory (LME), Faculty of Sciences, Ibn Zohr University, Agadir 80000, Morocco; bounalahcen@gmail.com (L.B.); a.benlhachemi@gmail.com (A.B.); 2 Department of Chemical, Environmental, and Materials Engineering, Higher Polytechnic School of Jaen, University of Jaen, Campus Las Lagunillas s/n, 23071 Jaen, Spain; rvl00010@red.ujaen.es; 3 Department of Industrial Engineering, University La Laguna, 38200 La Laguna, Spain; jmcacer@ull.edu.es; 4 Center for Advanced Studies in Earth Sciences, Energy and Environment (CEACTEMA), University of Jaen, Campus Las Lagunillas, s/n, 23071 Jaen, Spain; lperezvi@ujaen.es; 5 Department of Chemical, Environmental, and Materials Engineering, Higher Polytechnic School of Linares, University of Jaen, Campus Científico Tecnológico, Cinturón Sur s/n, 23700 Linares, Spain

**Keywords:** geopolymers, kaolinite, stainless steel slag, volcanic ash, mechanical properties

## Abstract

**Highlights:**

**What are the main findings?**

- Volcanic ash showed higher reactivity than stainless steel slag in geopolymer systems.
- 50MK–50VA achieved compressive strengths above 56 MPa after 28 days.

**What are the implications of the main findings?**

- Metakaolin (MK) was partially replaced by volcanic ash (VA) or stainless steel slag (SSS) in geopolymers.

**Abstract:**

The high environmental impact associated with ordinary Portland cement production has driven increasing interest in alternative low-carbon binder systems based on alkali-activated materials. In this context, geopolymers synthesized from metakaolin and supplemented with natural or industrial by-products represent a promising route toward more sustainable construction materials. In this study, the partial substitution of metakaolin (MK) with stainless steel slag (SSS, calcium rich) or volcanic ash (VA, silica-rich) in alkali-activated cements (AACs) synthesis was investigated by analyzing their physical, mechanical, and thermal properties. The structural evolution associated with alkali activation was assessed using X-ray diffraction (XRD) and ^29^Si and ^27^Al magic angle spinning nuclear magnetic resonance (MAS NMR). Fourier transform infrared spectroscopy (FTIR) revealed a shift in the main Si–O–T (T = Si, Al) asymmetric stretching band toward lower wavenumbers (≈1000 cm^−1^), indicating changes in the aluminosilicate network consistent with geopolymer formation. Scanning electron microscopy (SEM) was used to examine the microstructural features of the hardened matrices. The results showed that samples containing 50 wt.% MK and 50 wt.% VA achieved the highest mechanical performance, with compressive and flexural strengths of 46.29 MPa and 16.2 MPa at 7 days, increasing to 56.66 MPa and 17.58 MPa at 28 days of curing, respectively. In contrast, the samples containing 50 wt.% MK and 50 wt.% SSS displayed lower strength development, reaching compressive and flexural strengths of 27.7 MPa and 9.6 MPa at 7 days and 41.01 MPa and 13.68 MPa at 28 days. Additionally, thermal conductivity decreased with increasing porosity and decreasing bulk density, highlighting the potential of these AACs as structurally efficient materials with improved thermal insulation performance.

## 1. Introduction

The construction industry is one of the largest consumers of natural resources and a major contributor to global greenhouse gas emissions, primarily due to the extensive use of ordinary Portland cement (OPC) [1]. Cement production is an energy-intensive process involving high-temperature clinker formation and the calcination of limestone, both of which release significant amounts of carbon dioxide. It is estimated that cement manufacturing alone accounts for approximately 7% of global CO_2_ emissions, corresponding to nearly 2.5 gigatons of CO_2_ annually [2]. This substantial contribution exacerbates global warming and intensifies its associated impacts, including climate change, sea-level rise, and the increased frequency of extreme weather events. As global infrastructure demand continues to grow, reducing the environmental footprint of cementitious materials has become a critical priority for achieving sustainable development goals [3,4].

In response to increasing environmental concerns, regulatory pressure, and the urgent need to decarbonize the construction sector, alternative binder systems with reduced environmental impact have received considerable research attention. Among these alternatives, alkali-activated cements (AACs) have emerged as a promising class of sustainable binders due to their favorable mechanical performance, chemical durability, and significantly lower CO_2_ emissions compared to conventional OPC-based systems [5]. Alkali-activated cements (AACs) refer to cementitious binders produced by alkaline activation, while low-calcium systems within this group are specifically termed geopolymers. Geopolymers are inorganic aluminosilicate materials formed through alkali activation, in which silica- and alumina-rich precursors dissolve in alkaline media and subsequently undergo polycondensation reactions to form a three-dimensional amorphous or semi-crystalline network [6]. These materials are typically synthesized using industrial by-products or low-carbon precursors such as fly ash [7], electric arc furnace slags [8], and metakaolin [9], thereby enabling the valorization of waste materials while reducing dependence on virgin raw resources. As a result, AACs can exhibit excellent compressive strength, thermal stability, and durability [10], making them suitable for a wide range of applications, including structural concrete [11] and infrastructure exposed to aggressive environments [12,13].

Metakaolin (MK), obtained by the calcination of kaolinite-rich clays, is one of the most widely studied geopolymer precursors due to its high purity, amorphous structure, and consistent chemical composition [14]. The high reactivity of MK promotes efficient geopolymerization, resulting in dense microstructures and superior mechanical properties [5,15]. However, the production of MK requires thermal treatment, which increases energy consumption and cost. Consequently, partial substitution of MK with alternative natural or industrial waste-derived precursors has been increasingly explored to improve sustainability while maintaining desirable performance characteristics [16].

Volcanic ash (VA) is a pozzolanic material generated during volcanic eruptions [17]. It is a fine-grained, predominantly amorphous material composed of glassy particles with diameters typically below 2 mm [18]. Although its chemical composition depends on the type of volcano and eruption conditions, volcanic ash generally contains high proportions of silica (SiO_2_), alumina (Al_2_O_3_), and iron oxides (Fe_2_O_3_) [19]. The valorisation of volcanic ash as a raw material for construction applications represents a sustainable alternative for both the management of volcanic waste and the reconstruction of infrastructure damaged by volcanic events. Owing to its chemical composition and potential pozzolanic reactivity with Portland cement, VA can be used as a partial cement replacement (20–30 wt.%) in mortars and concretes, achieving satisfactory mechanical performance [20].

In addition to its use in blended cements, volcanic ash has been widely investigated as a precursor for alkali-activated materials and geopolymers. Tchakouté et al. [21] studied the alkaline activation of volcanic ash cured at ambient temperature using alkaline solutions with silica moduli ranging from 0.7 to 1.4, obtaining compressive strengths of up to 50 MPa. Djobo et al. [22] reported that, for structural applications requiring compressive strengths around 28 MPa, curing at 80 °C and a silica modulus of 1.40 are necessary for volcanic ash-based geopolymer synthesis. More recently, Tashima et al. [23] evaluated the alkaline activation of Cumbre Vieja volcanic ash, analyzing the effects of NaOH concentration and curing temperature. Their results demonstrated that compressive strengths up to 80 MPa can be achieved using appropriate alkali contents and curing at 65 °C.

Furthermore, Nana et al. [24] investigated the partial replacement of metakaolin with volcanic ash (10–30 wt.%) and the incorporation of reactive silica derived from rice husk ash (RHA, 0–20 wt.%). The authors reported optimum flexural and compressive strengths of 19.3 MPa and 60.73 MPa, respectively, for geopolymer composites containing 20 wt.% VA and 10 wt.% RHA, highlighting the synergistic effect of volcanic ash and supplementary reactive silica sources.

On the other hand, in 2022, global stainless steel production reached approximately 55.3 million metric tons [25]. During stainless steel manufacturing, between 200 and 500 kg of slag are generated per tonne of steel produced [26]. Stainless steel slags are primarily produced during two main stages: the electric arc furnace (EAF) process and the secondary refining stage, which includes Argon Oxygen Decarburization (AOD) and ladle metallurgy (LM) operations [27]. Currently, the utilization of stainless steel slag (SSS) remains limited, with most of the material being disposed of in landfills, leading to significant land occupation and potential environmental pollution [28]. Consequently, enhancing the valorisation of SSS is of considerable practical and environmental importance.

Stainless steel slag has been successfully applied as an aggregate in cement-based materials [29], in road pavement construction [30], and as a non-conventional supplementary cementitious material (SCM) in Portland cement systems [27]. However, SSS often contains free calcium oxide (CaO) and magnesium oxide (MgO), which exhibit expansive behavior and result in poor volumetric stability. This expansion can negatively affect the durability of concrete, thereby limiting the amount of SSS that can be safely incorporated as an SCM [31].

To overcome these limitations, several studies have explored the use of stainless steel slag as a precursor for alkali-activated materials (AAMs). Salman et al. [32] investigated the alkali activation of crystalline continuous-casting stainless steel slag using sodium and potassium silicate solutions combined with 5 M NaOH or KOH, under steam curing temperatures ranging from 60 to 110 °C. Their results demonstrated that both the type of alkali metal and the curing temperature significantly influence the strength development of alkali-activated slag systems. More recently, Ghorbani et al. [33] demonstrated that electric arc furnace stainless steel slag (EAFSS) can replace up to 50 wt.% of ground granulated blast-furnace slag (GGBFS), confirming its potential as an alternative precursor in the production of alkali-activated binders.

Despite the growing number of studies on metakaolin-based geopolymers incorporating either volcanic ash or metallurgical slags, several research gaps remain. Previous investigations have predominantly focused on a single alternative precursor or have emphasized mechanical performance alone, often without a comparative framework that considers natural and industrial by-products under identical synthesis conditions. In particular, a systematic comparison between silica-rich and calcium-rich aluminosilicate precursors as partial substitutes for metakaolin—integrating structural characterization, mechanical behavior, physical properties, and thermal performance—remains limited. Moreover, the influence of precursor substitution on microstructural development and its relationship with porosity and thermal conductivity has not yet been fully clarified.

In this context, the present study investigates volcanic ash (VA) and stainless steel slag (SSS) as two contrasting precursor families, representative of silica-rich and calcium-rich aluminosilicate materials, respectively. Geopolymers were synthesized by partially replacing metakaolin with VA or SSS under identical processing and curing conditions, enabling a direct and meaningful comparison of their effects on geopolymerization. The physical, mechanical, and thermal properties of the resulting materials were systematically evaluated, while the geopolymerization mechanisms and structural evolution were analyzed using X-ray diffraction (XRD), ^29^Si and ^27^Al MAS NMR, FTIR spectroscopy, and scanning electron microscopy (SEM). By emphasizing comparative trends rather than isolated results, this work provides new insights into how the chemical nature of sustainable precursor substitutions influences geopolymer performance and highlights the potential of both natural and industrial by-products for the development of environmentally responsible construction binders.

## 2. Materials and Methods

### 2.1. Raw Materials

The aluminosilicate source materials utilized in this study include natural kaolinite rich clay (Kao) sourced from Anzi village in Tiznit city, Morocco (29°28′19.3″ N 9°45′17.3″ W). To enhance its reactivity, the kaolinite rich clay underwent heat treatment at 600 °C for 3 h, resulting in the production of metakaolin (MK). The stainless-steel slag (SSS) originated from Acerinox S.A. industry situated in Los Barrios (Cádiz, Spain). The volcanic ash (VA) was obtained from eruption of the Cumbre Vieja volcano on the island of La Palma, Spain.

The SSS was initially crushed using a jaw crusher and then ground in a ball mill to obtain a fine powder. All precursor materials were passed through a 100 µm sieve to ensure uniform particle size, facilitate homogeneous mixing, and improve reactivity during the geopolymerization process.

Additionally, sodium hydroxide (NaOH) with a purity of 98% and sodium silicate (Na_2_SiO_3_) with a density of 1365 kg/m^3^ were utilized (PanReac AppliChem, Darmstadt, Germany). The composition of sodium silicate comprises H_2_O (61.9 wt.%), SiO_2_ (29.2 wt.%), and Na_2_O (8.9 wt.%).

### 2.2. Raw Materials Characterization

X-ray Fluorescence (XRF) is used to determine the chemical composition of the precursors MK, SSS, and VA determined with a Philips Magix Pro model PW-2440 equipment (Malvern, Worcestershire, UK). Table 1 presents the chemical composition of the precursors MK, SSS, and VA used in the preparation of the geopolymers. The oxide composition of these raw materials plays a crucial role in governing the geopolymerization reaction and the resulting physical and mechanical properties.

Metakaolin (MK) is characterized by a high silicon dioxide content (53.48 wt.%) and aluminum oxide (17.19 wt.%), along with moderate amounts of calcium oxide, iron oxide, and magnesium oxide, which collectively account for its high reactivity in alkaline environments. In contrast, stainless steel slag (SSS) exhibits a predominance of calcium oxide (43.40 wt.%), accompanied by notable contents of silicon dioxide and magnesium oxide. This calcium-rich composition strongly influences both the reaction mechanisms and the microstructural development of the resulting geopolymer matrix. Volcanic ash (VA), on the other hand, presents a heterogeneous chemical composition, with significant proportions of silicon dioxide, aluminum oxide, and iron oxide, together with appreciable amounts of calcium oxide and potassium oxide, which enhance its participation in the geopolymerization process.

**Figure 1:**
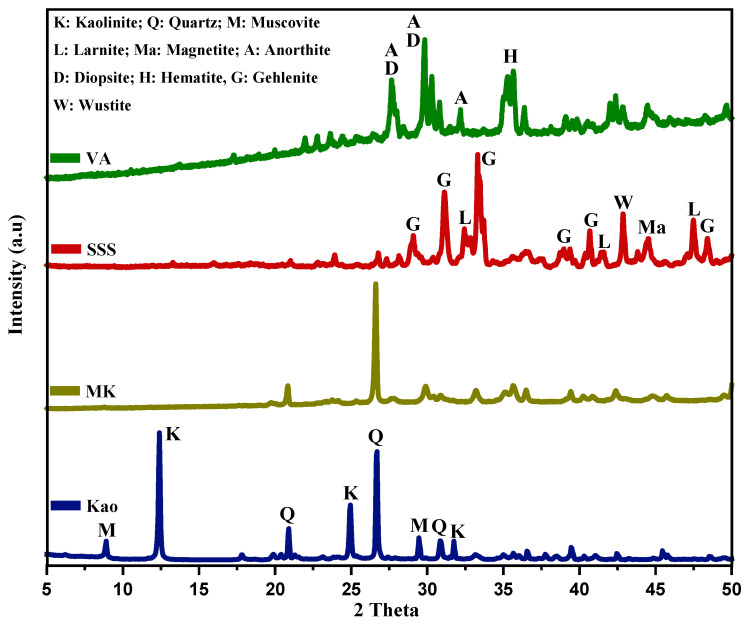
XRD patterns of raw materials.

The crystalline mineralogical phases of the raw materials were identified by X-ray diffraction (XRD) using an Empyrean diffractometer (Malvern PANalytical, Worcestershire, UK) equipped with a PIXcel-3D detector and Cu Kα radiation (λ = 1.5406 Å). Measurements were performed at an operating voltage of 40 kV and a current of 40 mA, over a 2θ range of 10–80°, with a step size of 0.02°. Phase identification was conducted using HighScore 5.2 software. Figure 1 shows the XRD diffractograms of the precursors. The XRD patterns of kaolin (Kao) exhibit characteristic diffraction peaks attributed to muscovite (KAl_2_(AlSi_3_O_10_)(OH)_2_, JCPDS 96-900-4642), kaolinite (Al_2_Si_2_O_5_(OH)_4_, JCPDS 80-0886), and quartz (SiO_2_, JCPDS 96-900-9667) [28]. After calcination, the XRD pattern of metakaolin (MK) shows the disappearance of kaolinite reflections, indicating a significant reduction in crystallinity due to structural disorder induced by high-temperature dehydroxylation. These observations are consistent with previously reported results [29,30].

The stainless steel slag (SSS) diffractogram reveals the presence of crystalline phases such as gehlenite (Ca_2_Al(SiAl)O_7_, JCPDS 35-0755), larnite (Ca_2_SiO_4_, JCPDS 90-2792), wüstite (FeO, JCPDS 01-1223), and magnetite (Fe_3_O_4_, JCPDS 19-0629). In contrast, the volcanic ash (VA) exhibits diffraction peaks corresponding to anorthite (CaAl_2_Si_2_O_8_, JCPDS 41-1486), diopside (CaMgSi_2_O_6_, JCPDS 41-1370), and hematite (Fe_2_O_3_, JCPDS 33-0664) [31,32].

Magic angle spinning nuclear magnetic resonance (MAS NMR) spectroscopy of ^27^Al and ^29^Si was employed to investigate the local structural environments of the precursor materials and the resulting geopolymers. The measurements were performed using a Unity Inova 300 spectrometer (Varian, Inc., Palo Alto, CA, USA) operating at a magnetic field strength of 7.05 T, corresponding to a proton resonance frequency of 300 MHz. The samples were packed into 4 mm zirconia rotors and spun at 10 kHz to minimize line-broadening effects. The ^27^Al chemical shifts were referenced to a 1 mol·L^−1^ aluminum nitrate solution at 0.00 ppm, while the ^29^Si spectra were referenced to Q_8_M_8_ at 11.45 ppm relative to tetramethylsilane (TMS).

**Figure 2:**
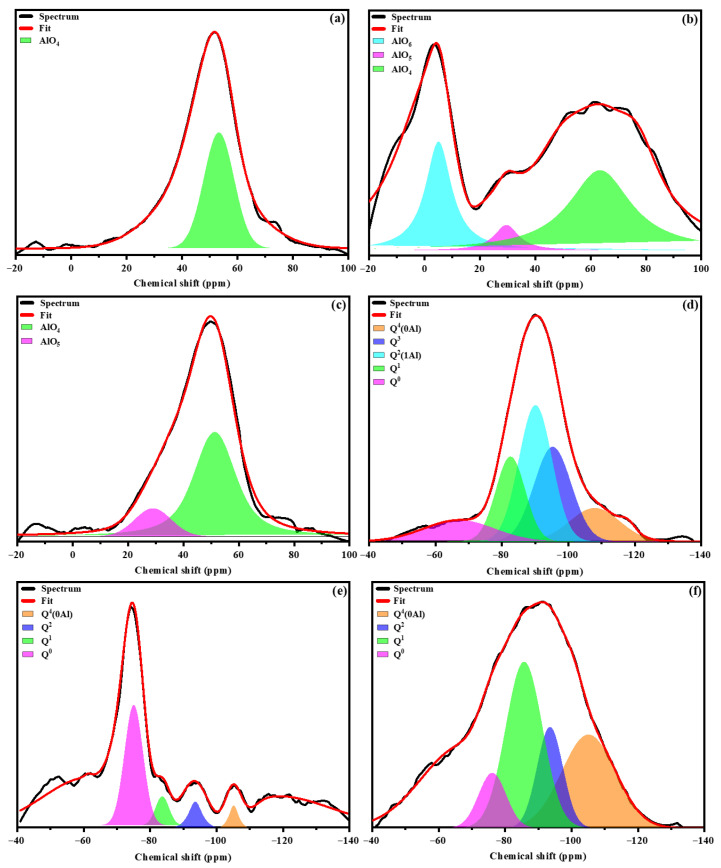
^27^Al (**a**,**b**,**c**) and ^29^Si (**d**,**e**,**f**) MAS NMR of precursors (MK (**a**,**d**), SSS (**b**,**e**), VA (**c**,**f**)).

The ^27^Al and ^29^Si MAS NMR spectra of the precursor materials are presented in Figure 2. The metakaolin (MK) spectrum (Figure 2a) exhibits a broad resonance centered at approximately 57 ppm, which is attributed to tetrahedrally coordinated aluminum (Al^IV^). The broad nature of this signal reflects the largely amorphous structure of metakaolin resulting from the dehydroxylation of kaolinite during calcination. Notably, the absence of a resonance associated with penta-coordinated aluminum (Al^V^) confirms the completeness of the calcination process. These results are consistent with previous findings reported by Ettahiri et al. [5].

In contrast, the stainless steel slag (SSS) spectrum (Figure 2b) displays three distinct resonances at approximately 60, 28, and 0 ppm, corresponding to tetrahedral (Al^IV^), pentahedral (Al^V^), and octahedral (Al^VI^) aluminum coordination environments, respectively [34]. The volcanic ash (VA) spectrum (Figure 2c) exhibits two resonances centered at around 50 and 26 ppm, indicating the presence of Al^IV^ and Al^V^ species [35].

To further elucidate the silicon coordination environments, Gaussian peak deconvolution of the ^29^Si MAS NMR spectra was conducted to identify the Q^4^(mAl) species (0 ≤ m ≤ 4), as shown in Figure 2d–f. Deconvolution of the precursor materials (MK, SSS, and VA) revealed resonances centered at approximately −107, −96, −90, −84, and −78 ppm, which are assigned to Q^4^, Q^3^, Q^2^, Q^1^, and Q^0^ silicate units, respectively [35]. The resonance observed at around −107 ppm is characteristic of fully polymerized Q^4^(0Al) silicon environments, typically associated with quartz and amorphous silica, in agreement with the XRD results.

**Figure 3:**
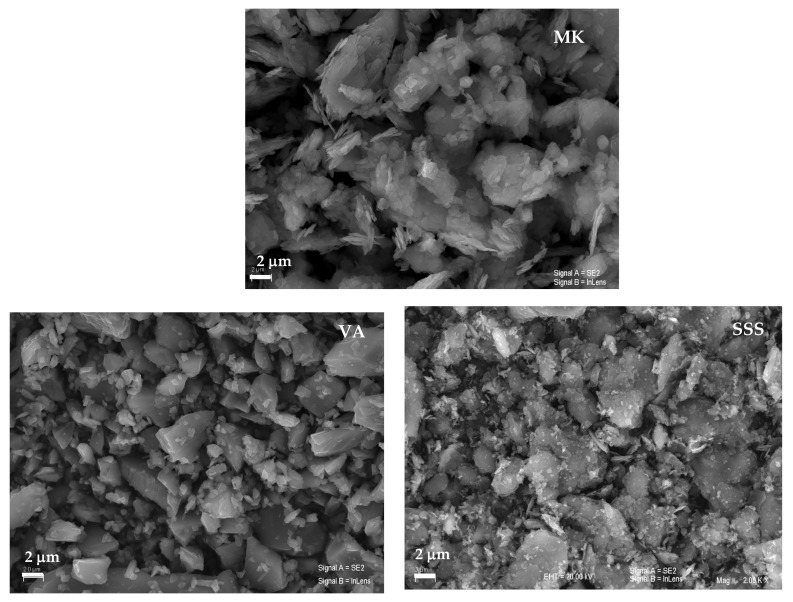
SEM micrographs of precursors.

Scanning electron microscopy (SEM) was employed to examine the morphological and microstructural characteristics of the raw materials and the resulting geopolymer samples. Observations were performed using a JEOL SM-840 scanning electron microscope (JEOL Ltd., Akishima, Tokyo, Japan), and representative micrographs are shown in Figure 3. The metakaolin (MK) precursor exhibits plate-like, sheet-structured particles, which are characteristic of the original kaolinite morphology [36]. In contrast, volcanic ash (VA) and stainless steel slag (SSS) present a heterogeneous particle size distribution, consisting of fine micrometer-sized particles together with larger, irregularly shaped particles featuring angular morphologies and sharp edges [37].

### 2.3. Alkali-Activated Cements (AACs) Preparation

The metakaolin (MK) and stainless steel slag (SSS) or MK and volcanic ash (VA) were combined in the dry state using a planetary kneader to ensure homogeneous mixing. Dry mixing was carried out for approximately 3 min. The alkaline solution was prepared in advance by dissolving the required amount of sodium hydroxide (NaOH) (10 M) in distilled water and addition of sodium silicate (Na_2_SiO_3_) under vigorous magnetic stirring until a homogeneous alkaline solution was obtained. The activating solution was allowed to cool to room temperature before being gradually added to the solid precursor mixture. Mixing was continued for 5 min until a homogeneous and workable fresh geopolymer paste was obtained. As summarized in Table 2, the composition of the alkaline activator and the liquid-to-solid ratio were adjusted according to the type and proportion of the precursor materials. This strategy was adopted to account for differences in chemical composition, intrinsic reactivity, and water demand among MK, VA, and SSS. The simultaneous adjustment of these parameters ensured adequate workability, homogeneous mixing, and effective geopolymerization across all formulations.

The fresh geopolymer pastes were cast into stainless steel molds to produce prismatic specimens with dimensions of 1 × 1 × 6 cm^3^. The molds were placed on a vibrating table to remove entrapped air and subsequently sealed with plastic film to prevent moisture loss. The specimens were cured at 60 °C for 24 h, a temperature selected to accelerate the geopolymerization reaction and promote sufficient early-age strength development, particularly in mixtures containing less reactive precursors. After demolding, all specimens were stored at room temperature until testing at curing ages of 7 and 28 days.

**Figure 4:**
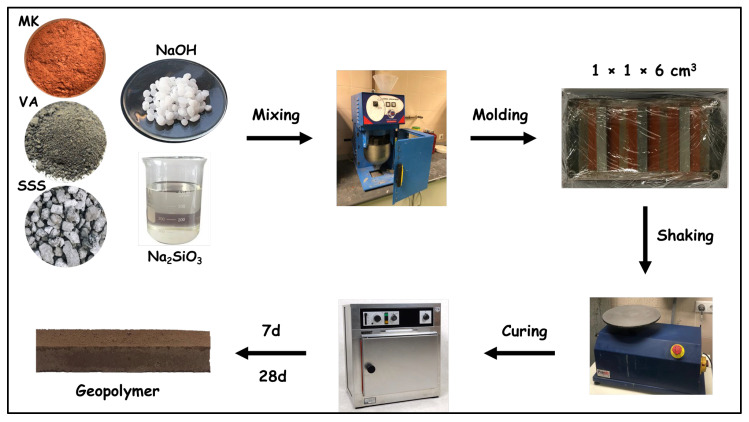
Preparation protocol of AAM samples.

A schematic illustration of the AAMs preparation procedure is presented in Figure 4, and the detailed mix proportions are reported in Table 2.

### 2.4. Characterization Methods

Compressive and flexural strength tests were conducted to evaluate the mechanical performance of the AACs specimens in accordance with the UNE-EN 1015-11:2000/A1:2007 standard [38]. Flexural strength was measured using an MTS Insight 5 testing machine with a 5 kN load capacity at a displacement rate of 0.2 mm/min, while compressive strength tests were performed on an MTS 8101 universal testing machine with a capacity of 100 kN. The standard UNE-EN 1936:2007 [39] was used to determine the bulk density, apparent porosity, and water absorption of specimens according to the Archimedes principle. The thermal conductivity of 55 mm diameter and 15 mm height specimens after 28 days of curing was determined using a FOX 50 TA instruments heat flow meter, according to ISO 8302 [40]. Attenuated Total Reflection Fourier Transform Infrared Spectroscopy (ATR-FTIR) was performed using a Vertex 70 Bruker in the range 400–4000 cm^−1^. The same analytical equipment and operating conditions described for the characterization of the raw materials were also employed for the analysis of the hardened geopolymer samples. X-ray diffraction (XRD), ^27^Al and ^29^Si magic angle spinning nuclear magnetic resonance (MAS NMR), and scanning electron microscopy (SEM) measurements were conducted using identical instruments, experimental settings, and analytical protocols to ensure consistency and comparability between the precursor materials and the resulting geopolymer matrices.

## 3. Results and Discussion

### 3.1. XRD of AACs

**Figure 5:**
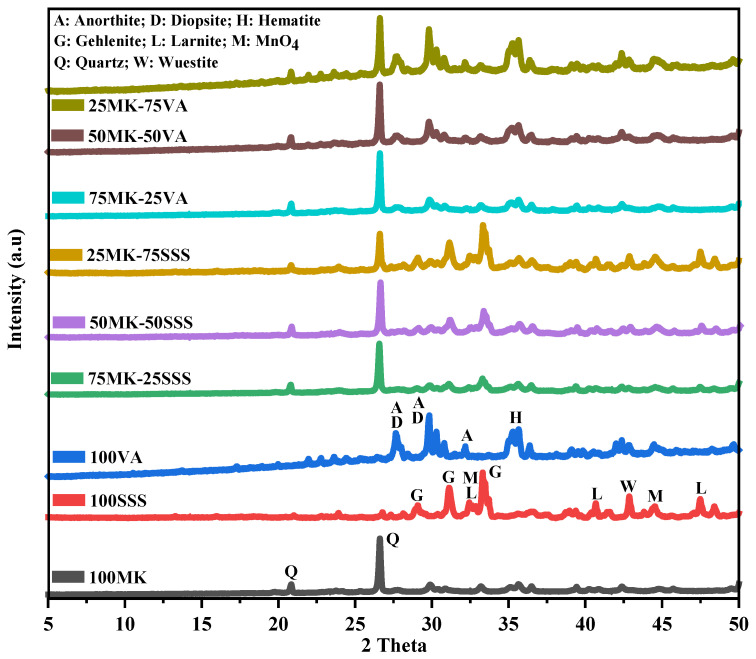
XRD of AACs.

Figure 5 presents XRD diffractograms of AACs. The X-ray diffraction patterns of the AACs reveal a predominantly amorphous structure, as indicated by the broad hump observed between 20° and 35° 2θ, which is characteristic of the formation of an aluminosilicate network during geopolymerization [41]. Compared with the raw precursors, many of the sharp crystalline peaks from metakaolin, volcanic ash (VA), and stainless steel slag (SSS) are attenuated, reflecting partial dissolution of the precursor phases. Residual crystalline peaks, such as those associated with quartz and calcium-rich phases from SSS, are still detectable, indicating that some unreacted material remains in the hardened matrix. AACs with higher VA content display a more pronounced amorphous hump, consistent with efficient geopolymerization due to the silica- and alumina-rich nature of the volcanic ash. In contrast, AACs with higher SSS content exhibit sharper peaks corresponding to residual calcium-rich phases, suggesting the partial formation of calcium-aluminosilicate hydrates (C–A–S–H) and a lower proportion of amorphous N–A–S–H gel. These observations demonstrate that the chemical composition of the precursor, silica-rich versus calcium-rich, significantly influences the degree of amorphization and the balance between amorphous and crystalline phases in the final binder, which in turn affects the mechanical and thermal properties of the materials.

### 3.2. MAS-NMR (^29^Si and ^27^Al) Analysis

**Figure 6:**
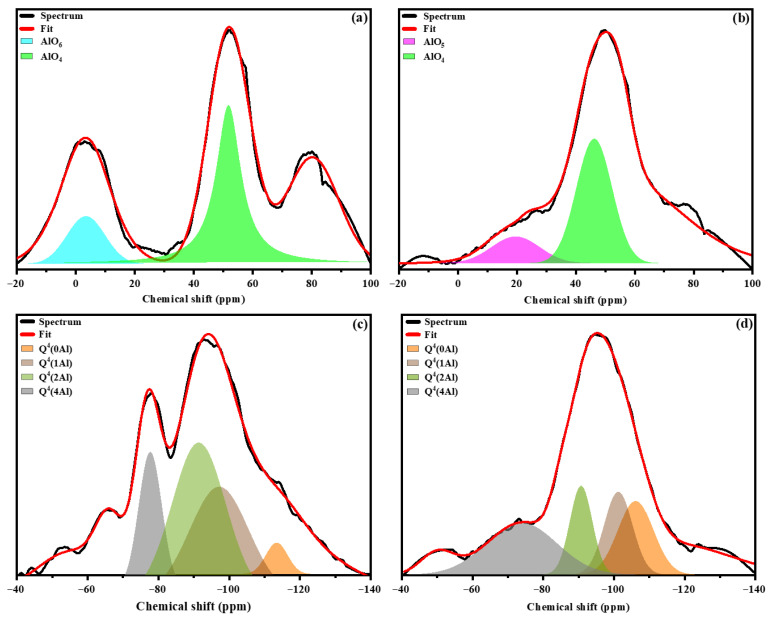
^27^Al (**a**,**b**) and ^29^Si (**c**,**d**) MAS NMR of geopolymers (50MK-50SSS (**a**,**b**), 50MK-50VA (**c**,**d**)).

To gain further insight into the short-range structural organization of the hardened binders, the experimental and deconvoluted ^27^Al and ^29^Si MAS NMR spectra of the geopolymer samples are presented in Figure 6. After geopolymerization, clear differences in aluminum and silicon environments are observed depending on the nature of the secondary precursor.

The ^27^Al MAS NMR spectrum of the 50MK–50SSS geopolymer displays two main resonances centered at approximately 50 and 0 ppm, assigned to tetrahedrally coordinated Al^IV^ and octahedrally coordinated Al^VI^, respectively. The persistence of a significant Al^VI^ contribution suggests that part of the aluminum remains in calcium-stabilized environments, which are commonly associated with the formation of calcium-rich reaction products or with the incomplete incorporation of aluminum into the three-dimensional aluminosilicate framework. This behavior reflects the influence of the calcium-rich SSS on the geopolymerization mechanism, which tends to promote the coexistence of geopolymeric gel with Ca-containing phases rather than a fully polymerized N–A–S–H-type network [42].

In contrast, the 50MK–50VA specimen is characterized by a dominant resonance around 48 ppm, attributed to Al^IV^, and a weak signal near 18 ppm corresponding to Al^V^. The predominance of tetrahedrally coordinated aluminum indicates a higher degree of aluminum incorporation into the aluminosilicate network, consistent with the formation of a well-developed three-dimensional geopolymer structure. The minor presence of Al^V^ is commonly reported in highly polymerized aluminosilicate systems and is often interpreted as an intermediate coordination state within the amorphous geopolymer gel [22,43]. This structural configuration highlights the more effective participation of the silica-rich VA in the geopolymerization process.

Further insight into the silicate network connectivity is provided by the ^29^Si MAS NMR spectra. Compared to the precursors, both AACs formulations exhibit a systematic shift in the ^29^Si resonances toward lower frequencies (more negative chemical shifts), indicative of an increased degree of polymerization and the formation of more condensed Q^4^ units. These shifts reflect the incorporation of aluminum into the second coordination sphere of silicon, resulting in Q^4^(mAl) species with higher Al substitution.

Notably, the presence of Q^4^(2Al) and Q^4^(3Al) units in both AACs confirms the development of an amorphous aluminosilicate framework characteristic of geopolymer gels. However, the relative distribution of these units suggests a more homogeneous and highly cross-linked network in the 50MK–50VA system, whereas the calcium-rich environment in 50MK–50SSS likely disrupts the continuity of the aluminosilicate network, leading to a more heterogeneous gel structure [42].

### 3.3. FTIR Analysis

**Figure 7:**
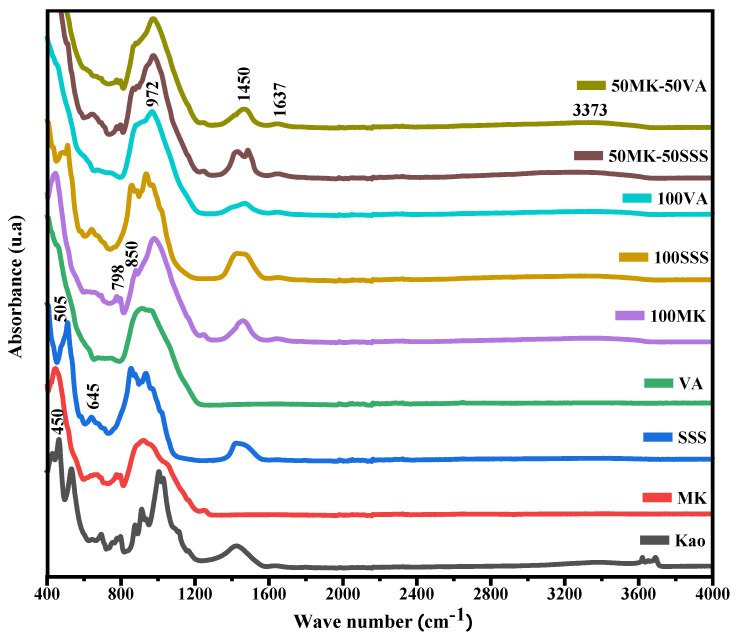
FTIR spectra of precursors and AACs.

Figure 7 presents the FTIR spectra of the precursor materials and the synthesized geopolymers, allowing evaluation of the structural evolution associated with alkali activation and the influence of the chemical nature of the incorporated residues. The band observed at approximately 1637 cm^−1^ is attributed to the bending vibrations of H–O–H groups associated with physically adsorbed water, while the signal located around 3673 cm^−1^ corresponds to O–H stretching vibrations [44]. These hydroxyl-related bands disappear in the metakaolin sample after calcination at 600 °C, confirming the dehydroxylation of kaolinite and the formation of an amorphous and highly reactive aluminosilicate phase [45].

In the spectra of kaolinite, the stainless steel slag (SSS), and the geopolymer samples, a band centered around 1450 cm^−1^ is observed and assigned to the asymmetric stretching vibrations of O–C–O bonds in carbonate species. This feature indicates partial atmospheric carbonation, promoted by the presence of free alkali species (Na^+^ and K^+^) in the matrix, which react with atmospheric CO_2_ to form carbonates or bicarbonates [46]. This phenomenon is particularly relevant in calcium-rich systems containing SSS, where the formation of calcium carbonates may be enhanced.

The main absorption band associated with the asymmetric stretching of Si–O–T (T = Si or Al), located near 1000 cm^−1^, shows a noticeable shift toward lower wavenumbers after geopolymerization. This shift is widely recognized as a fingerprint of the alkali activation process and reflects the structural reorganization of the system, associated with the dissolution of precursor glassy phases and the subsequent formation of a new aluminosilicate network [33]. In geopolymers incorporating the silica-rich volcanic ash (VA), this shift suggests a higher degree of polymerization of the silicate framework and the development of a more continuous and highly condensed network, consistent with the predominance of a N–A–S–H type gel. In contrast, in SSS-containing systems, the high calcium content modifies the network structure, favoring the coexistence of aluminosilicate domains with calcium-rich phases and resulting in a more heterogeneous matrix.

Additionally, a band observed around 850 cm^−1^ is associated with the stretching vibrations of Ca–O and Si–O bonds, characteristic of calcium-containing phases such as larnite, and is particularly evident in the SSS-based formulations [47]. The persistence of this band indicates that part of the calcium-rich phases does not fully dissolve during alkali activation and remains as residual or partially reactive components within the hardened matrix. The band detected near 798 cm^−1^ is attributed to Si–O stretching vibrations of quartz, evidencing the presence of residual crystalline silica, which is more pronounced in VA-containing systems due to their high silica content [48]. Finally, the bands located at approximately 505 and 450 cm^−1^ are assigned to the bending vibrations of O–Si–O and Si–O bonds, respectively, typical of aluminosilicate structures [49].

### 3.4. SEM-EDS Study

**Figure 8:**
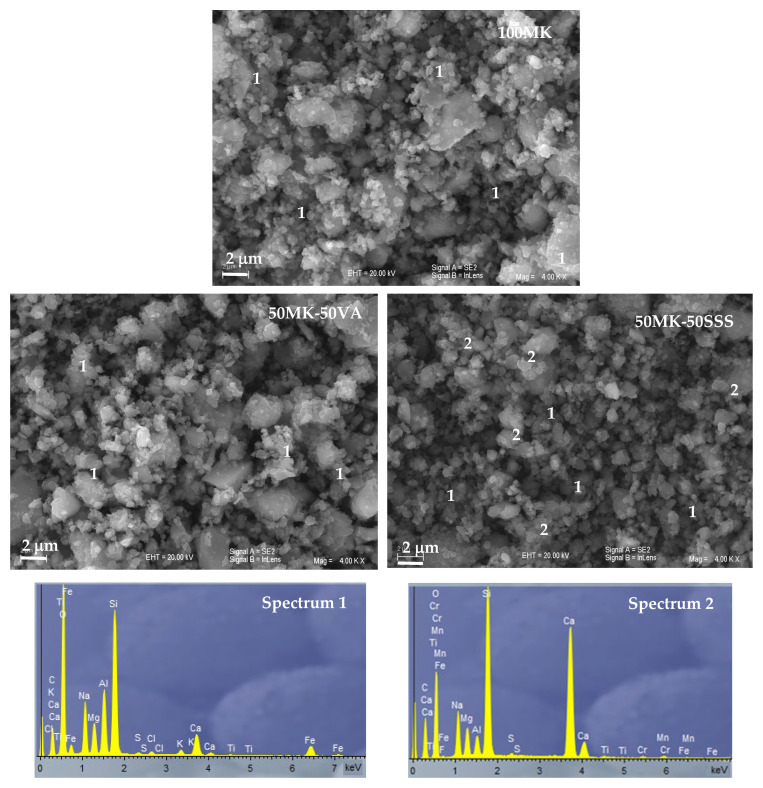
SEM-EDS analysis of 100 MK, 50MK-50VA and 50MK-50SSS AACs.

Figure 8 presents the SEM micrographs of 100 MK, 50MK-50VA and 50MK-50SSS AACs. The SEM micrograph of the 100MK AAC shows the formation of a relatively homogeneous and compact binding matrix, characteristic of metakaolin-based low-calcium alkali-activated systems. The microstructure is dominated by a continuous gel phase with few distinguishable unreacted particles, indicating an efficient dissolution of the metakaolin precursor and the development of a well-connected aluminosilicate network. EDS analysis of this matrix confirms a Si–Al-Na-rich composition with minor amounts of calcium, consistent with the formation of a typical aluminosilicate gel (Spectrum 1).

The alkali-activated materials incorporating volcanic ash (50MK-50VA) exhibit a microstructure comparable to that of the 100MK system, although with a slightly more heterogeneous appearance due to the presence of partially reacted ash particles. The matrix remains continuous and cohesive, with reaction products embedding residual crystalline phases derived from the volcanic ash. EDS spectra indicate a predominance of silicon, aluminum and sodium with limited calcium contribution, reflecting the silica-rich nature of the volcanic ash and supporting the formation of aluminosilicate-dominated binding phases similar to those observed in the metakaolin-based system (Spectrum 1).

In contrast, the alkali-activated cement containing stainless steel slag (50MK-50SSS) shows a more heterogeneous microstructure, characterized by the presence of partially reacted slag particles embedded within the binding matrix. While a continuous gel phase is still observed, SEM–EDS analyses reveal clear compositional variations within the matrix. Certain regions exhibit elevated calcium contents, as evidenced by Spectrum 2, indicating localized formation of calcium-enriched reaction products, whereas other areas display Si–Al-Na-dominated compositions comparable to those of the 100MK and VA-based systems (Spectrum 1). This coexistence of calcium-rich and aluminosilicate-rich regions highlights the chemical heterogeneity of the binding phase in slag-containing AACs and reflects the influence of the calcium-rich precursor on the alkali-activation process.

### 3.5. Compressive and Flexural Strengths

Mechanical performance, particularly compressive and flexural strength, is a key criterion for assessing the suitability of AACs binders for structural and construction applications.

**Figure 9:**
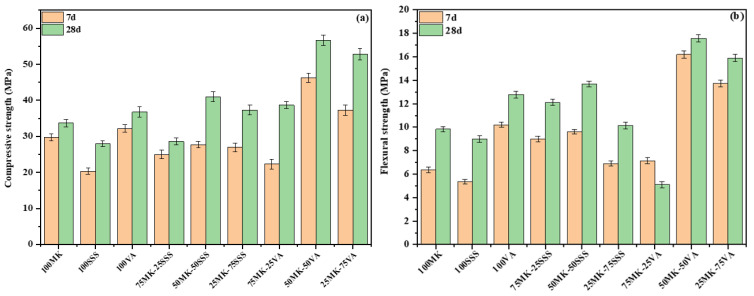
Compressive (**a**) and flexural strength (**b**) of AACs.

Figure 9 shows the evolution of compressive strength as a function of precursor type and curing time. AACs synthesized from single precursors (100MK, 100SSS, and 100VA) exhibit distinct strength development trends. After 7 days of curing, compressive strengths of 29.7 MPa, 20.33 MPa, and 32.2 MPa were obtained for 100MK, 100SSS, and 100VA, respectively, increasing to 33.65 MPa, 27.92 MPa, and 36.74 MPa after 28 days. The comparatively lower strength achieved by the stainless steel slag-based AACs reflects the reduced reactivity of SSS under alkaline activation, which can be attributed to its high crystallinity, elevated calcium content, and limited availability of reactive amorphous aluminosilicate phases.

Clear differences in mechanical behavior are observed when comparing blended AACs systems containing volcanic ash or stainless steel slag. AACs incorporating volcanic ash consistently exhibit superior mechanical performance compared to those containing stainless steel slag (VA > SSS), highlighting the strong influence of precursor chemistry on geopolymer gel formation and network development. Volcanic ash, characterized by a high content of reactive silica and alumina and a relatively low calcium concentration, readily dissolves under alkaline conditions and promotes the formation of a well-developed, highly polymerized aluminosilicate network. Among all formulations, the 50MK–50VA AACs exhibits the highest mechanical performance, achieving compressive and flexural strengths of 46.29 MPa and 16.2 MPa after 7 days, which further increase to 56.66 MPa and 17.58 MPa after 28 days of curing. The enhanced performance of this system is attributed to the synergistic effect between metakaolin and the silica-rich volcanic ash, which favors extensive geopolymer gel formation and results in a dense and homogeneous microstructure. In addition, the presence of iron species in volcanic ash may contribute to gel development by participating in condensation reactions and facilitating the formation of a more interconnected aluminosilicate network [22].

Previous studies support these observations. Djobo et al. [50] demonstrated that volcanic ash-based geopolymers can achieve high early-age strength under optimized synthesis conditions, particularly at elevated curing temperatures. Similarly, Hossain [19] reported that while increasing volcanic ash or pumice content in blended cement systems may reduce compressive strength due to cement dilution, the high silica content of these materials enables pozzolanic reactions that contribute to long-term strength development. Moreover, Bawab et al. [51] successfully produced a cement-free binder using volcanic ash and calcium carbide residue, achieving a compressive strength of 31.2 MPa after 28 days, further confirming the high reactivity and mechanical potential of silica-rich volcanic materials.

In contrast, AACs incorporating stainless steel slag exhibit comparatively lower strength development. The reduced mechanical strength can be primarily attributed to its high crystallinity, which limits the dissolution of reactive silicon (Si) and aluminum (Al) species under alkaline activation conditions. In contrast, calcium species present in SSS readily dissolve in the alkaline medium due to the high CaO content of the residue. The dissolution of Ca^2+^ ions promotes interactions with Si–O–Si and Al–O–Si bonds, leading to the preferential formation of calcium-rich reaction products such as C–A–S–H gel or hybrid (C,N)–A–S–H gels rather than a pure N–A–S–H geopolymer structure. Although these Ca-bearing gels may contribute to early-age strength, their formation often results in a heterogeneous and less cross-linked microstructure, which ultimately limits the development of long-term mechanical performance. The 50MK–50SSS AACs achieves compressive and flexural strengths of 27.7 MPa and 9.6 MPa after 7 days, increasing to 41.01 MPa and 13.68 MPa after 28 days, respectively. Although these values are suitable for certain construction applications, they remain lower than those obtained for VA-containing systems.

Consistent with this behavior, Qiao et al. [27] reported cementitious materials produced from stainless steel slag with a compressive strength of 28.36 MPa after 28 days of curing, comparable to the strength levels typically achieved in SSS-based alkali-activated systems. These results highlight the combined influence of high crystallinity, limited Si and Al dissolution, and preferential calcium-driven gel formation on the mechanical performance of SSS-containing AACs. Ghorbani et al. [33] observed a reduction in mechanical performance with increasing steel slag content in alkali-activated materials. This reduction was attributed to changes in microstructural evolution and reduced binder densification associated with the predominance of calcium-rich phases.

For all formulations, a progressive increase in both compressive and flexural strength is observed with extended curing time, indicating the continued evolution of the reaction products and ongoing structural reorganization within the binder matrix. This strength gain is associated with the gradual dissolution of reactive phases and the progressive condensation of aluminosilicate species, leading to the densification of the geopolymer network.

### 3.6. Physical Properties

**Figure 10:**
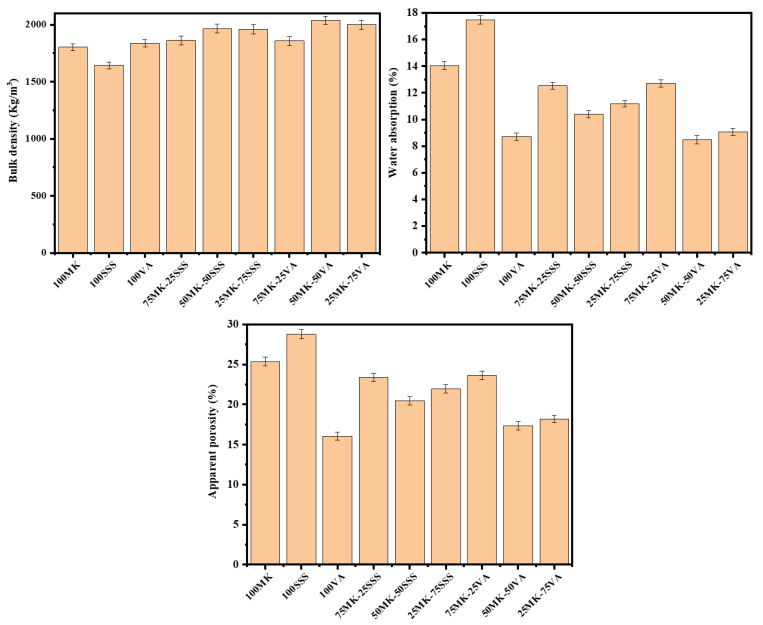
Bulk density, apparent porosity and water absorption of AACs.

Figure 10 presents the bulk density, apparent porosity, and water absorption values of the AACs after 28 days of curing. Clear differences in physical properties are observed among the systems prepared with single precursors. The bulk densities of 100MK, 100SSS, and 100VA are 1803, 1643, and 1835 kg/m^3^, respectively. These values are in good agreement with the corresponding apparent porosity and water absorption results, with the SSS-based geopolymer exhibiting the lowest density and the highest porosity. This behavior can be attributed to the high crystallinity and calcium-rich nature of stainless steel slag, which limits the dissolution of reactive phases during alkali activation and leads to a less compact microstructure.

For the blended systems, the geopolymer prepared with a 50MK–50VA ratio exhibits the highest bulk density (2038 kg/m^3^), together with relatively low apparent porosity (8.48%) and water absorption (17.32%). This enhanced densification is associated with the silica-rich composition of volcanic ash, which promotes the formation of a highly polymerized aluminosilicate network (N–A–S–H gel), resulting in a denser and more homogeneous matrix. In contrast, the 50MK–50SSS geopolymer shows a lower bulk density (1966 kg/m^3^) and higher apparent porosity (10.39%) and water absorption (10.46%). In this case, the high calcium content of SSS favors the formation of calcium-containing reaction products, such as C–A–S–H or hybrid (C,N)–A–S–H gels, which tend to produce a more heterogeneous pore structure.

Overall, an increase in bulk density is associated with a reduction in apparent porosity and water absorption, which correlates well with the observed improvement in mechanical strength. These trends are consistent with the findings of Gómez-Casero et al. [52], who investigated the incorporation of black steel slag (BSS) into biomass bottom ash (BBA) as a precursor for alkali-activated materials. Their results showed that bulk density increases with higher BSS content, higher curing temperatures, and longer curing times. For a mixture containing 50% BBA and 50% BSS, a bulk density of 1687 kg/m^3^ was reported, which is lower than the values obtained in the present study, highlighting the beneficial role of metakaolin and precursor reactivity in promoting matrix densification.

### 3.7. Thermal Conductivity Analysis

**Figure 11:**
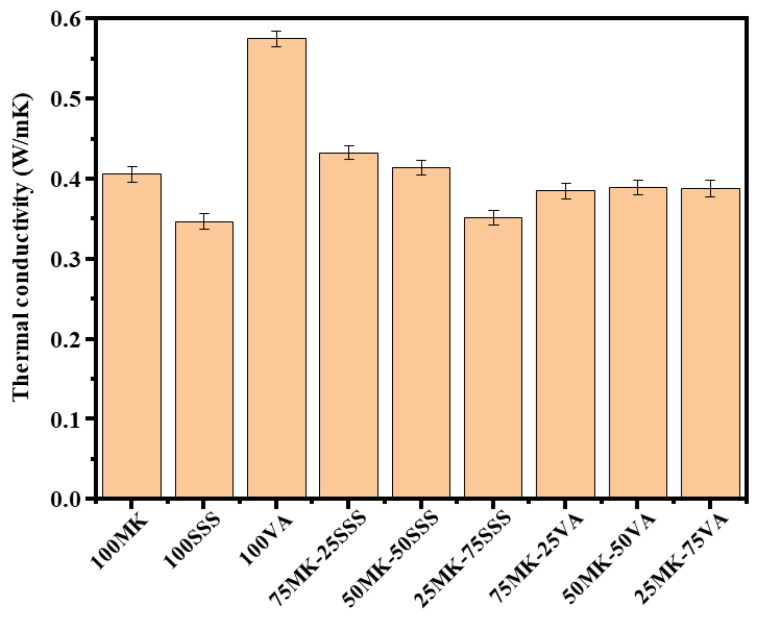
Thermal conductivity of AACs after 28 days of curing.

Figure 11 presents the thermal conductivity values of the AACs after 28 days of curing. The measured thermal conductivities are 0.406 W/m·K for 100MK, 0.347 W/m·K for 100SSS, and 0.575 W/m·K for 100VA. A clear influence of precursor composition on thermal behavior is observed. In particular, increasing the proportion of stainless steel slag in the mixture leads to a progressive reduction in thermal conductivity, from 0.433 to 0.352 W/m·K. This trend is mainly associated with the higher porosity and lower bulk density of SSS-containing systems, which promote increased phonon scattering and reduced heat transfer.

Conversely, increasing the volcanic ash content results in a slight increase in thermal conductivity, from 0.385 to 0.388 W/m·K. This behavior is attributed to the silica-rich nature of volcanic ash, which favors the formation of a denser and more continuous aluminosilicate network, thereby facilitating heat conduction. Overall, the results indicate that thermal conductivity decreases with increasing porosity and decreasing bulk density, highlighting the strong influence of geopolymer microstructure on heat transfer properties.

All AACs formulations exhibit significantly lower thermal conductivity than conventional Portland cement, which typically shows values around 1.5 W/m·K [53]. This marked reduction, combined with the adequate mechanical strength and relatively low density of the AACs, enhances their suitability for applications requiring both structural performance and thermal insulation. Furthermore, the superior insulating properties of these materials make them particularly attractive for energy-efficient building applications, contributing to reduced energy consumption and improved indoor thermal comfort.

## 4. Conclusions

This study demonstrates the feasibility of partially replacing metakaolin with both volcanic ash (VA) and stainless steel slag (SSS) in AACs, contributing to the valorization of natural and industrial by-products within low-carbon and sustainable construction materials. The main conclusions drawn from the study are:

1. The combined use of X-ray diffraction, ^27^Al and 29 Si MAS NMR spectroscopy, FTIR and SEM-EDS analysis confirmed the successful formation of geopolymeric reaction products in all formulations, while revealing clear differences in structural organization depending on the chemical nature of the secondary precursor.
2. Volcanic ash, characterized by its silica-rich composition and higher proportion of reactive amorphous phases, exhibited greater reactivity under alkaline activation than stainless steel slag. This enhanced reactivity promoted the development of a highly polymerized aluminosilicate network, leading to superior mechanical performance and a more homogeneous microstructure in VA-containing AACs.
3. In contrast, stainless steel slag, with its high calcium content and predominantly crystalline phases, showed limited dissolution under the applied activation conditions. The presence of abundant Ca^2+^ ions favored the formation of calcium-rich reaction products, such as C–A–S–H and hybrid (C,N)–A–S–H gels, resulting in a more heterogeneous matrix and comparatively lower strength development.
4. The physical properties of the AACs were strongly influenced by precursor composition. VA-containing systems exhibited higher bulk density and lower apparent porosity, reflecting a more compact microstructure, whereas SSS-containing geopolymers displayed increased porosity associated with incomplete precursor dissolution and calcium-driven phase assemblages.
5. Thermal conductivity was found to be closely related to microstructural features, decreasing with increasing porosity and decreasing bulk density. Thermal conductivity values ranged from 0.347 to 0.575 W/m·K, which are significantly lower than those of conventional Portland cement, highlighting their potential for applications requiring both mechanical reliability and enhanced thermal insulation.

Overall, under the investigated synthesis and curing conditions, volcanic ash proved to be a more effective supplementary precursor than stainless steel slag in metakaolin-based AACs. Its silica-rich composition enables a favorable balance between mechanical performance, matrix densification, and thermal efficiency, while stainless steel slag remains a viable alternative for applications where moderate strength and improved insulation properties are prioritized.

## Figures and Tables

**Table 1:** Chemical composition of precursors.

Oxides (wt.%)	SiO_2_	Al_2_O_3_	CaO	Fe_2_O_3_	K_2_O	MgO	SO_3_	Na_2_O	Cr_2_O	MnO	P_2_O_5_	TiO_2_	LOI
MK	53.48	17.19	10.79	8.24	1.79	4.26	0.45	1.83	-	-	0.16	-	1.81
SSS	30.20	5.75	43.40	2.01	0.01	11.30	-	0.02	2.84	1.61	0.01	1.25	1.60
VA	43.82	14.15	11.11	13.15	1.69	7.08	0.13	4.05	0.06	0.20	0.92	3.46	0.18

**Table 2:** Mix proportions of geopolymers samples series.

Sample	MK (g)	SSS (g)	VA (g)	NaOH (g)	Na_2_SiO_3_ (g)	H_2_O (g)	Liquid/Solid
100MK	200	-	-	18.26	117	44.09	0.9
100SSS	-	200	-	8.12	52	19.23	0.4
100VA	-	-	200	10.14	65	24.21	0.5
75MK-25SSS	150	50	-	16.23	104	39.12	0.8
50MK-50SSS	100	100	-	14.2	91	34.15	0.7
25MK-75SSS	50	150	-	12.17	78	29.18	0.6
75MK-25VA	150		50	16.23	104	39.12	0.8
50MK-50VA	100	-	100	14.2	91	34.15	0.7
25MK-75VA	50	-	150	12.17	78	29.18	0.6

## Data Availability

The original contributions presented in this study are included in the article. Further inquiries can be directed to the corresponding authors.

## Abbreviations

The following abbreviations are used in this manuscript:

MK	Metakaolin
VA	Volcanic Ash
SSS	Stainless Steel Slag
Kao	Kaolinite rich clay
OPC	Ordinary Portland Cement
AACs	Alkali-Activated Cements
AAMs	Alkali-Activated Materials
XRD	X-ray Diffraction
XRF	X-ray Fluorescence
FTIR	Fourier Transform Infrared Spectroscopy
ATR-FTIR	Attenuated total reflectance-Fourier Transform Infrared Spectroscopy
SEM	Scanning Electronic Microscopy
EDS	Energy Dispersive X-ray Spectroscopy
MAS NMR	Magic Angle Spinning Nuclear Magnetic Resonance
LOI	Loss on Ignition
TMS	Tetramethylsilane
